# Supplementary material for: Prognostic and Immunological Role of STK38 across Cancers: Friend or Foe?
Source: Int J Mol Sci. 2022 Sep 30;23(19):11590. doi: 10.3390/ijms231911590 (PMC9570386; doi:10.3390/ijms231911590)
Supplement: Supplementary file 1 [file ijms-23-11590-s001.zip › ijms-1902668-supplementary.pdf]

Figure S1 Phylogenetic tree of *STK38*. A constraint-based multiple alignment tool of NCBI was used to obtain the phylogenetic tree of STK38 in different species.

Figure S2 Correlation between *STK38* gene expression and prognosis of cancers using the Kaplan-Meier plotter. Kaplan-Meier plotter was used to conduct a series of survival analyses, including OS, DMFS, RFS, PFS, PPS and FP in breast cancer (A), gastric cancer (B), lung cancer (C), liver cancer (D) and ovarian cancer (E) cases.

Figure S3 Correlation between *STK38* expression and DNA methylation level in cervical squamous cell carcinoma (CESC), colon adenocarcinoma (COAD), esophageal carcinoma (ESCA), rectum adenocarcinoma (READ), thyroid carcinoma (THCA), uterine carcinosarcoma (UCS), pheochromocytoma and paraganglioma (PCPG), testicular germ cell tumors (TGCT), thymoma (THYM).

Figure S4 Radar map illustrating the correlation between *STK38* expression and tumor mutational burden (TMB). The red lines represent correlation coefficients, and blue values represent ranges. Spearman correlation test, $*p < 0.05$, $**p < 0.01$, $***p < 0.001$.

Figure S5 Radar map illustrating the relationship between *STK38* expression and microsatellite instability (MSI). The red lines represent correlation coefficients, and blue values represent ranges. Spearman correlation test, $*p < 0.05$, $**p < 0.01$, $***p < 0.001$.

Figure S1

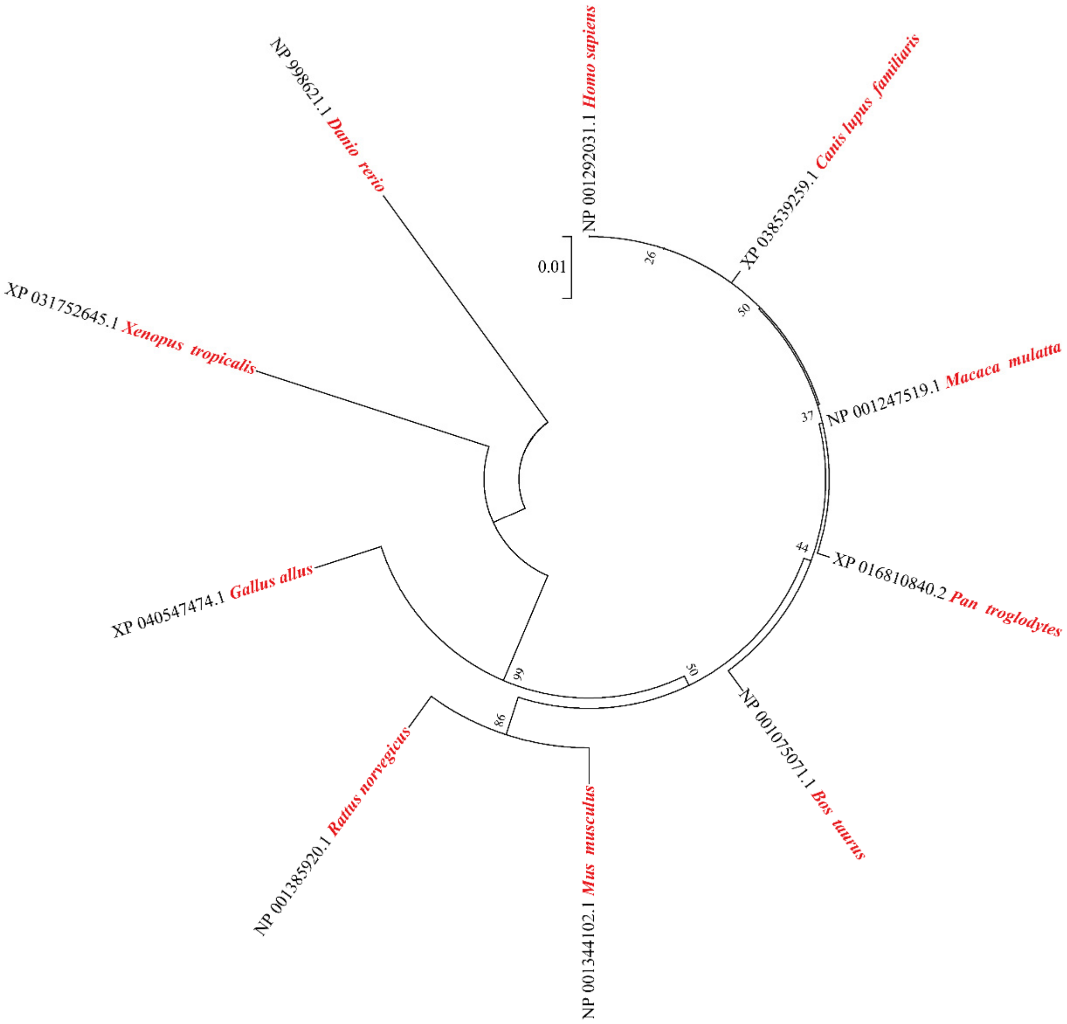

NP 001292031.1 Homo sapiens
XP 038539259.1 Canis lupus familiaris
NP 001247519.1 Macaca mulatta
XP 016810840.2 Pan troglodytes
NP 001075071.1 Bos taurus
NP 001344102.1 Mus musculus
NP 001385920.1 Rattus norvegicus
XP 040547474.1 Gallus allus
XP 031752645.1 Xenopus tropicalis
NP 998621.1 Danio rerio
0.01
26
50
37
44
50
99
86

Figure S2

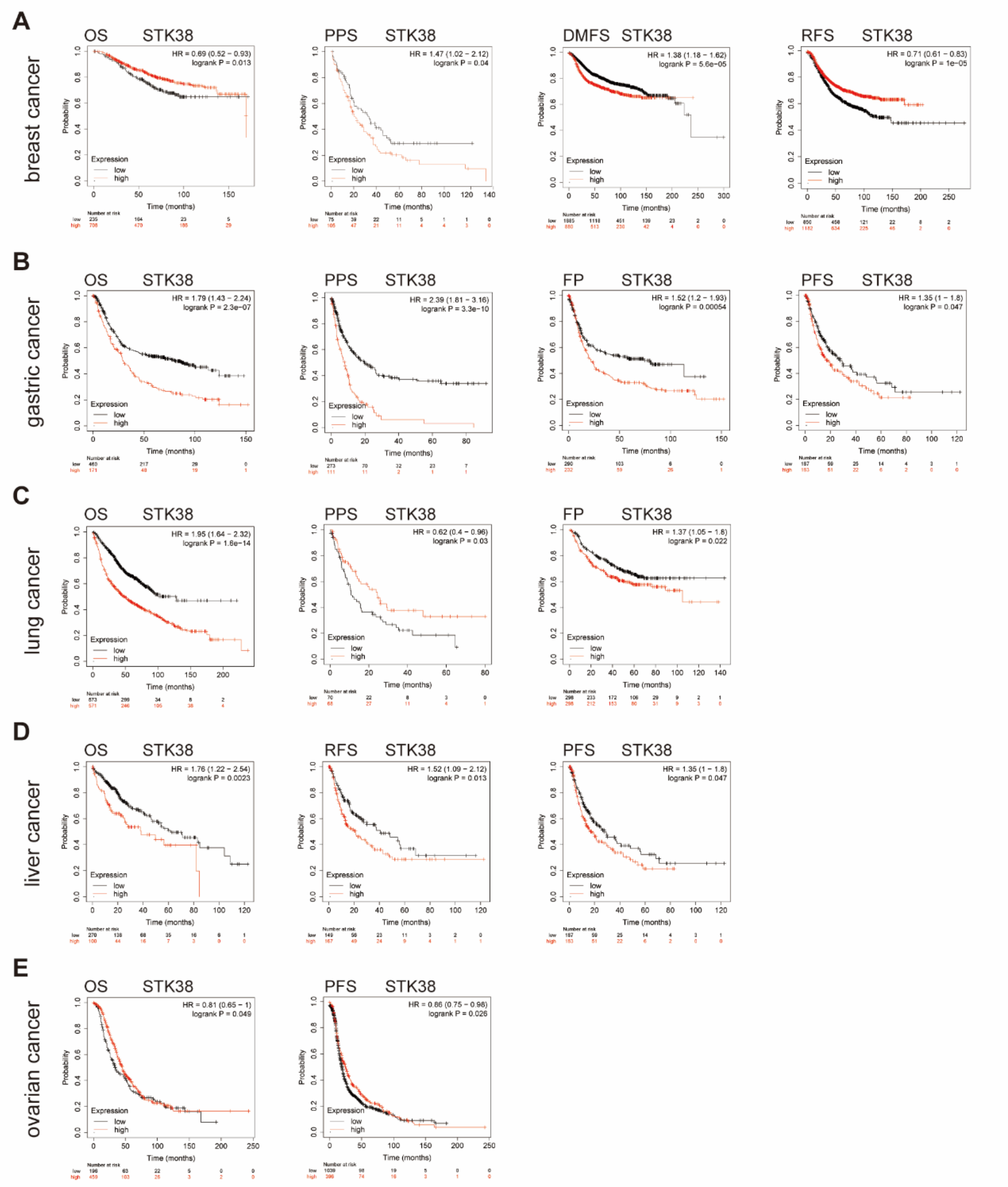

A
breast cancer
OS STK38
HR = 0.69 (0.52 − 0.93)
logrank P = 0.013
PPS STK38
HR = 1.47 (1.02 − 2.12)
logrank P = 0.04
DMFS STK38
HR = 1.38 (1.18 − 1.62)
logrank P = 5.6e−05
RFS STK38
HR = 0.71 (0.61 − 0.83)
logrank P = 1e−05
B
gastric cancer
OS STK38
HR = 1.79 (1.43 − 2.24)
logrank P = 2.3e−07
PPS STK38
HR = 2.39 (1.81 − 3.16)
logrank P = 3.3e−10
FP STK38
HR = 1.52 (1.2 − 1.93)
logrank P = 0.00054
PFS STK38
HR = 1.35 (1 − 1.8)
logrank P = 0.047
C
lung cancer
OS STK38
HR = 1.95 (1.64 − 2.32)
logrank P = 1.6e−14
PPS STK38
HR = 0.62 (0.4 − 0.96)
logrank P = 0.03
FP STK38
HR = 1.37 (1.05 − 1.8)
logrank P = 0.022
D
liver cancer
OS STK38
HR = 1.76 (1.22 − 2.54)
logrank P = 0.0023
RFS STK38
HR = 1.52 (1.09 − 2.12)
logrank P = 0.013
PFS STK38
HR = 1.35 (1 − 1.8)
logrank P = 0.047
E
ovarian cancer
OS STK38
HR = 0.81 (0.65 − 1)
logrank P = 0.049
PFS STK38
HR = 0.86 (0.75 − 0.98)
logrank P = 0.026
Probability
Time (months)
Expression
low
high
Number at risk

Figure S3

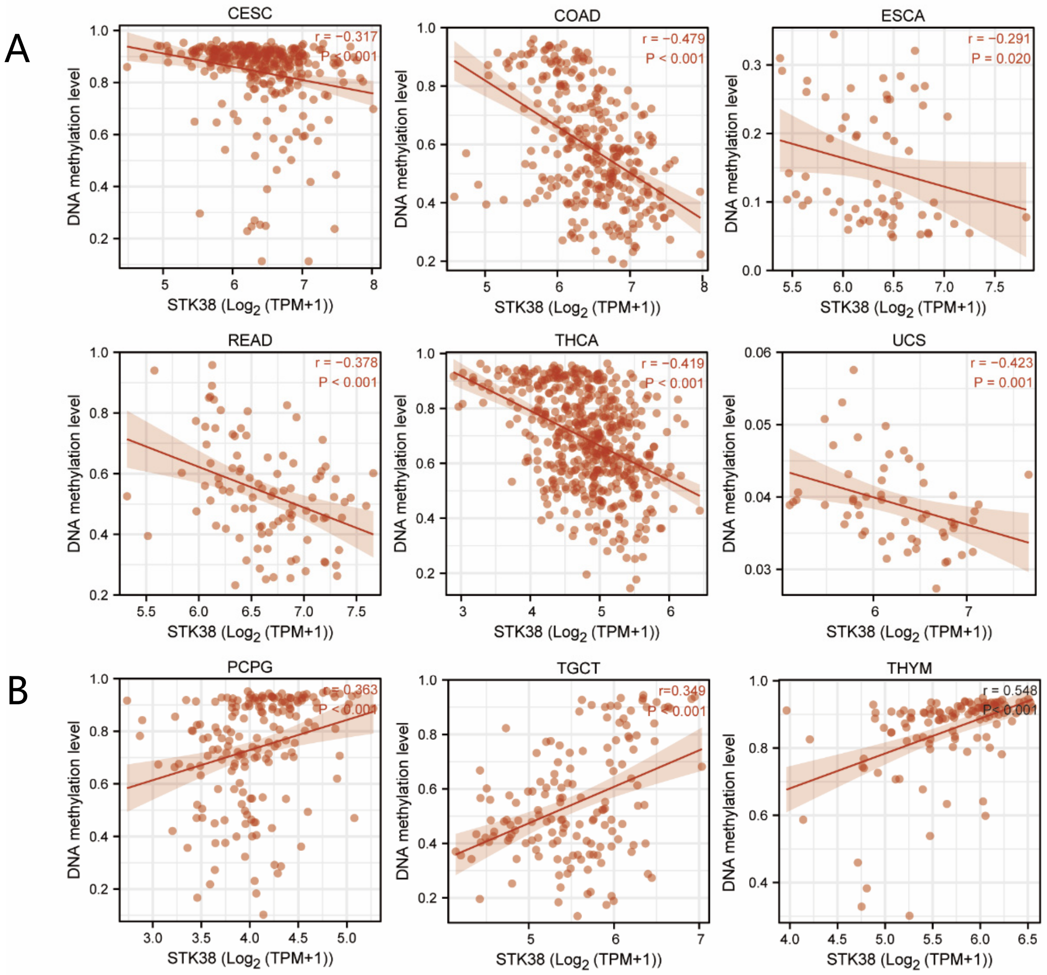
A
CESC
r = −0.317
P < 0.001
COAD
r = −0.479
P < 0.001
ESCA
r = −0.291
P = 0.020
READ
r = −0.378
P < 0.001
THCA
r = −0.419
P < 0.001
UCS
r = −0.423
P = 0.001
B
PCPG
r = 0.363
P < 0.001
TGCT
r=0.349
P < 0.001
THYM
r = 0.548
P< 0.001
DNA methylation level
STK38 (Log2 (TPM+1))

Figure S4

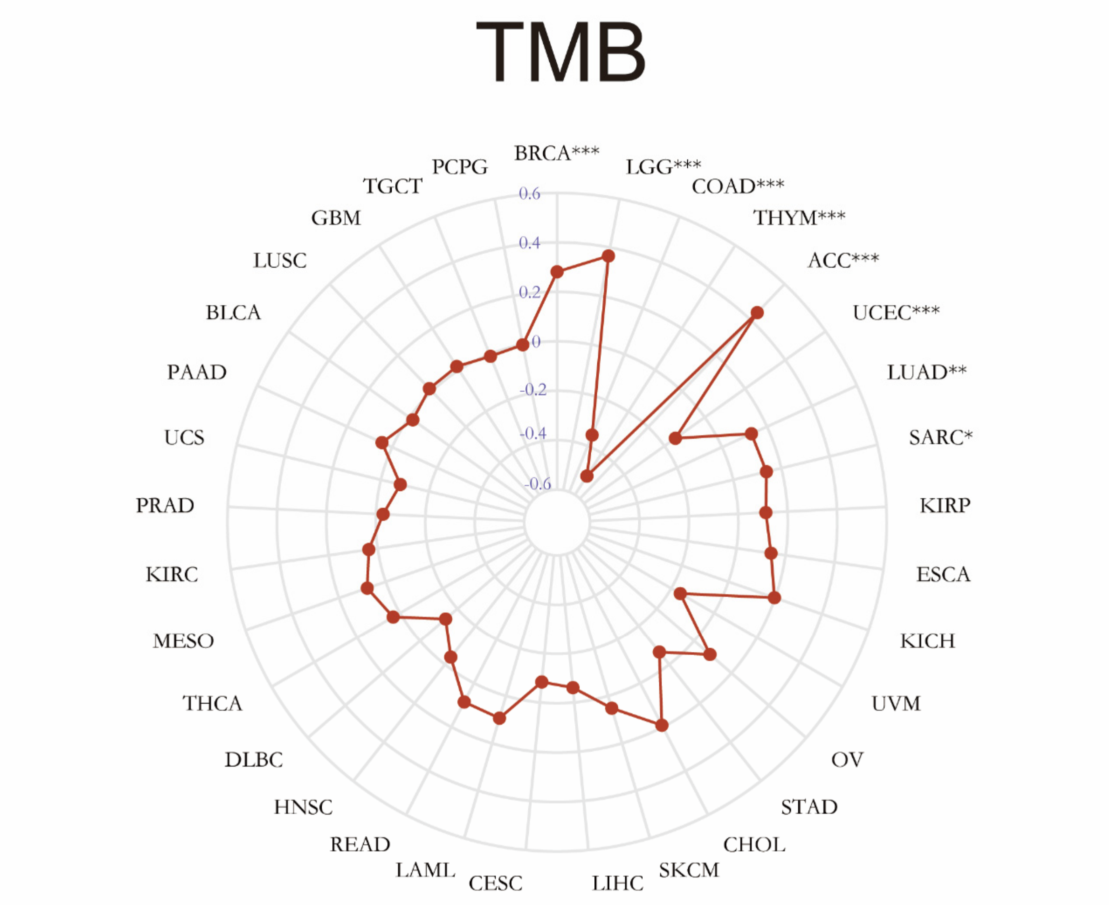
TMB
BRCA***
LGG***
COAD***
THYM***
ACC***
UCEC***
LUAD**
SARC*
KIRP
ESCA
KICH
UVM
OV
STAD
CHOL
SKCM
LIHC
CESC
LAML
READ
HNSC
DLBC
THCA
MESO
KIRC
PRAD
UCS
PAAD
BLCA
LUSC
GBM
TGCT
PCPG
0.6
0.4
0.2
0
-0.2
-0.4
-0.6

Figure S5

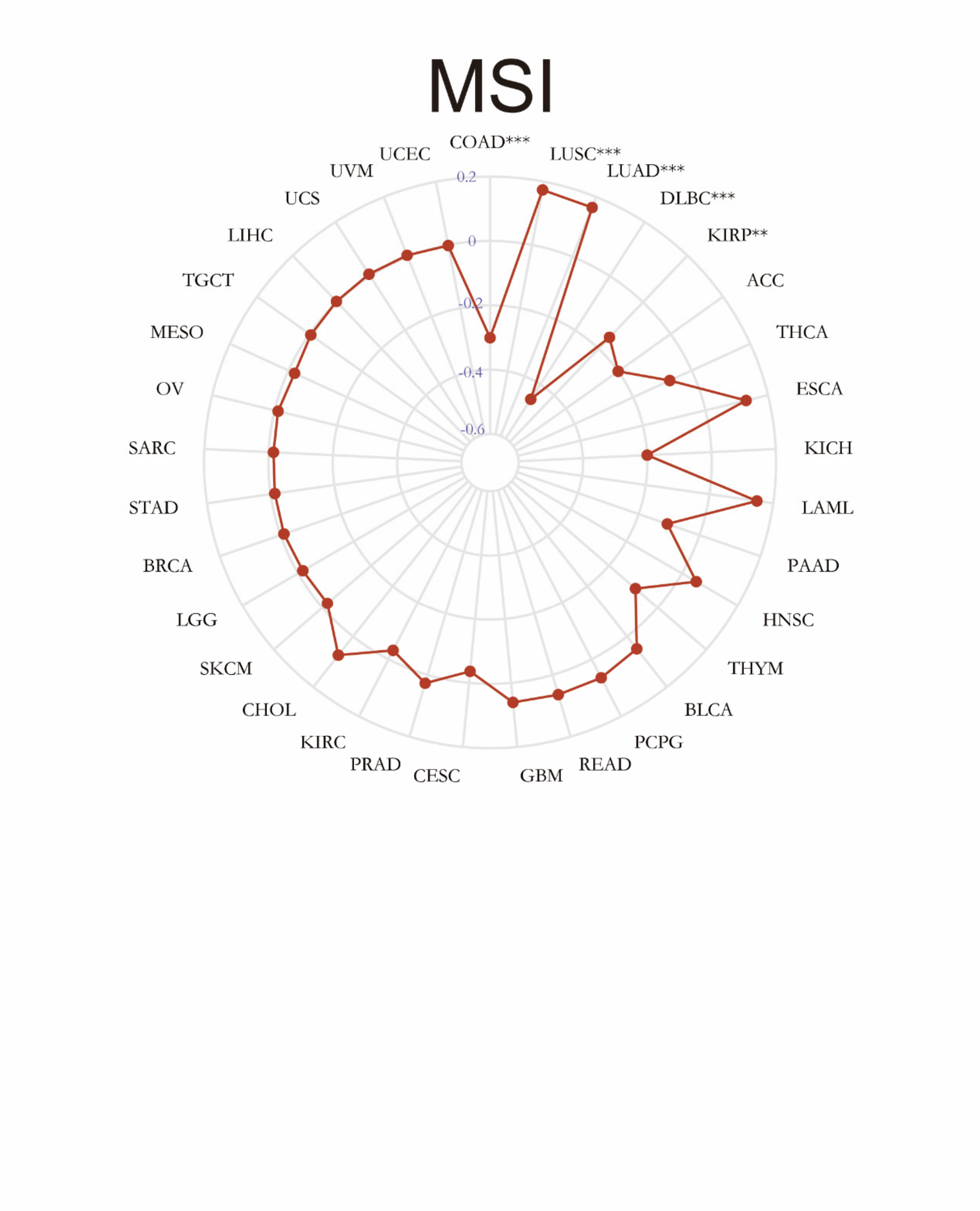
MSI
COAD***
LUSC***
LUAD***
DLBC***
KIRP**
ACC
THCA
ESCA
KICH
LAML
PAAD
HNSC
THYM
BLCA
PCPG
READ
GBM
CESC
PRAD
KIRC
CHOL
SKCM
LGG
BRCA
STAD
SARC
OV
MESO
TGCT
LIHC
UCS
UVM
UCEC
0.2
0
-0.2
-0.4
-0.6
